# Jaw osteonecrosis management around a dental implant inserted 2 years before starting treatment with zoledronic acid

**DOI:** 10.4317/jced.52234

**Published:** 2015-07-01

**Authors:** Ana-Belén Marín-Fernández, Blas García Medina, Antonio Aguilar-Salvatierra, Alberto Jiménez-Burkhardt, Gerardo Gómez-Moreno

**Affiliations:** 1 Oral and Maxillofacial Surgery Service, Virgen de las Nieves University Hospital, Granada, Spain; 2 Pharmacological Research in Dentistry Group, Master in Periodontology and Implant Dentistry, Faculty of Dentistry, University of Granada, Granada, Spain; 3 Director of Pharmacological Research in Dentistry Group and Master of Periodontology and Implant Dentistry, Department of Special Care in Dentistry, Faculty of Dentistry, University of Granada, Granada, Spain

## Abstract

Bisphosphonates (BP) are a type of drug known to inhibit bone resorption through complex interventions. Their primary mechanism of action is aimed at the cellular level, inhibiting osteoclast activity and so bone resorption. BPs are widely used, with many patients receiving continuous treatment for years. But it is well known that these drugs can produce osteonecrosis of the jaw (ONJ). Zoledronic acid (ZA) is an intravenous BP used in the treatment and prophylaxis of bone disease in patients with malignant tumors with bone implication. ZA is the most potent BP in clinical development. This report describes the case of a 62-year-old woman with breast cancer antecedents which relapsed, who had received a maxillary dental implant two years before the start of therapy with zoledronic acid. She later developed osteonecrosis of the jaw (ONJ), which began in the peri-implant area, and was treated for stage 3 ONJ by sub-total maxillectomy.

** Key words:**Bisphosphonates, zoledronic acid, osteonecrosis of the jaw, peri-implantitis, maxillectomy.

## Introduction

Bisphosphonates (BP) are a type of drug known to inhibit bone resorption through complex mechanisms. Their primary action is aimed at the cellular level, inhibiting osteoclast activity and so bone resorption, particularly when administered by intravenous infusion. Because some breast cancer treatments can cause bone loss (osteoporosis), many women in treatment for breast cancer are also prescribed a BP (1).

Osteonecrosis of the jaw (ONJ) is an oral complication of bisphosphonate medication. Bisphosphonate-related ONJ will include a previous history of bisphosphonate receipt, bone exposure, and absence of radiotherapy to the maxilla. The strength of the bisphosphonate, duration of exposure, and an antecedent of bone trauma, condition ONJ development. Zoledronic acid (ZA) is a bisphosphonate used in the treatment and prophylaxis of bone disease in patients with malignant tumors with bone implication. ZA is the most potent BP in clinical development. The potential anticancer activity of zoledronic acid (ZA) may be mediated through its effects on the bone marrow microenvironment. In addition, preclinical evidence suggests that ZA treatment interferes directly or indirectly with other processes in cancer progression and tumor growth (2).

This article describes the case of a female patient with zoledronic acid-related ONJ with extensive maxillary affectation in the area where an implant had been placed 2 years before starting treatment with zoledronic acid. The case was resolved by left-side subtotal maxillectomy that included part of the nasal fossa floor and nasal mucosa and the anterior maxillary sinus wall and floor.

## Case Report

A 62-year-old woman came to the Oral and Maxillofacial Surgery clinic at *Virgen de las Nieves* University Hospital (Granada, Spain) with an intraoral lesion of several months’ evolution. As a medical antecedent, it was noted that the patient had been diagnosed with breast cancer in 1993 and treated with surgery, chemotherapy, and radiotherapy. In 2005 she suffered a relapse of the cancer which was again treated with surgery, chemotherapy, and radiotherapy. At the end of 2010 she was diagnosed with bone metastasis in the right supra-acetabular iliac blade, and was prescribed adjuvant drug treatment with intravenous ZA (1 dose per month for 14 months).

The patient had received dental implant treatment 2 years before starting treatment with ZA, which consisted of one implant at 24 (which was missing; all teeth were present in the rest of the dental arch) and implants to replace teeth 14 and 16. Before the oral lesion appeared, the patient was being treated for peri-implantitis in the region of the implant at 24. She had suffered discomfort since the beginning of 2011 in the left maxillary premolar region. In September 2011, extraction of 25 was performed, which presented percussion pain and associated pathology, while the implant at 24 was maintained. Antibiotic treatment with oral doxycycline, 500 mg once a day for 1 month, was prescribed in December 2011 as the onset of ONJ was suspected. As symptoms persisted, the patient attended regular check-ups but no infection focus or evident bone necrosis were observed and the patient continued peri-implantitis treatment. In October 2013, the patient presented implant mobility at 24 with associated infection and areas of bone necrosis.

**Figure 1:**
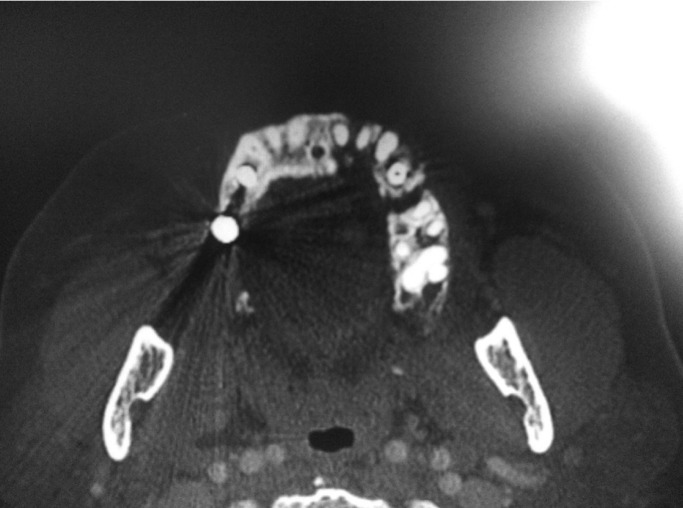
CT shows left side maxillary osteonecrosis.

Clinical exploration identified an extensive ulcer in the left maxilla with bone exposure (necrotic bone exposure of the alveolar bone at the implant site at 24), but computerized tomography (CT) revealed that necrosis extended from 21 to 25 (although bone exposure had only occurred in the area of 24). The suspected diagnosis was of a lesion compatible with bisphosphonate-related osteonecrosis. Associated with this lesion, the patient also presented an intraoral fistula with purulent drainage. An orthopantomograph and facial CT were made (Fig.1), which confirmed the diagnosis of stage 3 bisphosphonate-related ONJ (3).

**Figure 2:**
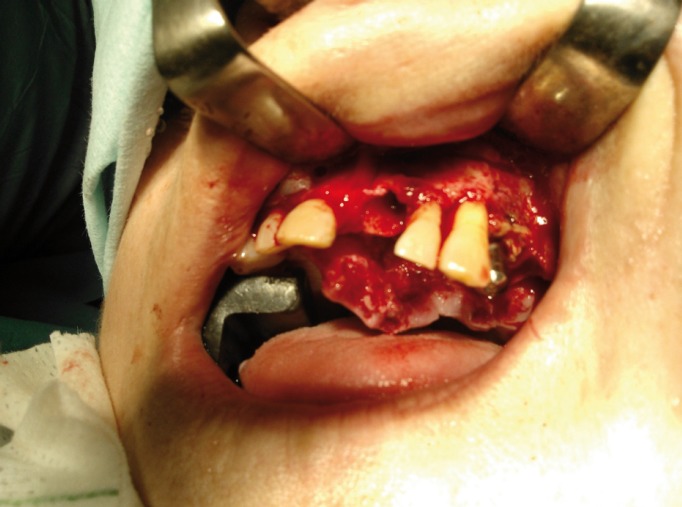
Surgical procedure of maxillary necrosis resection.

In February 2014, after several days in hospital, when mouth washing with a 0.12% chlorhexidine gluconate solution was prescri-bed every 8 hours (Perio Aid, Dentaid, Barcelona, Spain) and intravenous antibiotic treatment with amoxicillin+calvulanic acid (Augmentin IV powder solution injected 1 g/200mg every 8 hours) for managing the superinfection, it was decided to perform radical surgery of the ONJ area. Under general anesthetic subtotal maxillectomy was performed from 21 to 26 including the entire osteonecrosis area, the floor of the nasal fossa and mucosa, and anterior floor and wall of the maxillary sinus (Fig. 2). Bone defect coverage was performed with direct closure by gingiva and vestibular mucosa. Pathological anatomy of the surgically-removed piece confirmed ONJ diagnosis. Antibiotic treatment continued for a further 10 days after surgery, together with 0.12% chlorhexidine mouth washing for a further 2 months. The patient returned for a check-up every 2 weeks to monitor the case’s evolution. Ten months after surgery, the patient was seen to be in clinical and radiographic remission.

## Discussion

Patients with breast cancer are often treated intravenously with ZA or other BPs to suppress osteoclast activity as in the classic case described here (2). As many studies have suggested, patients who receive intravenous BP therapy along with additional chemotherapy and corticosteroids are at a high risk of ONJ (4,5).

In this way, for patients in treatment with bisphosphonates, the current objective of case management is ONJ prevention (6). It is essential to find out if a patient is or has been in treatment with bisphosphonates; this requires exhaustive exploration of medical histories, given that patients will not always remember what medication they were prescribed years earlier. The health professional must know what bisphosphonate the patient is taking or has taken, the route of administration (as this conditions the risk of developing ONJ as intravenous bisphosphonates lead to a higher risk of ONJ), for how long, the indication, and frequency.

In recent years, the number of cases of ONJ following dental implant insertion in patients treated with bisphosphonates has been increasing (7). Importantly, ONJ may appear in a peri-implant area, particularly when implants have been placed some time before starting bisphosphonate therapy as in the present case (implants placed 2 years before BP administration). For this reason, it would appear advisable that patients who are or have been in treatment by bisphosphonates and also have dental implants should undergo a check-up every 6 months, as peri-implantitis can be a warning signal for the onset of ONJ. Furthermore, the patient should be warned when considering dental implant therapies that if he/she is taking or is going to take bisphosphonates, then regular check-ups are advisable. The patient should give informed consent in writing to the effect that he/she understands that implant failure is a possibility as well as ONJ in the peri-implant area.

The main approach to treating ONJ are the management of symptoms, infection, and to prevent new areas of ONJ from developing, and are conditioned by ONJ stage (3). Stage 1 requires conservative treatment; stage 2 local and systemic antibiotic treatment, chlorhexidine mouthwashes, and local debridement where necessary; stage 3, which presents extensive areas of necrosis and infection, will need radical surgery rather than local debridement (as in the present case) but in no case is reconstruction with autologous bone or vascularized bone grafting indicated.

Physicians must be informed and trained to deal with this new clinical entity, in order to establish prophylactic care, early diagnosis, and to prevent the potentially devastating consequences (8). Moreover, close coordination between the general practitioner prescribing BP and the dentist is essential for reducing the risk of BP-related ONJ in patients starting BP therapy (9).
